# Curation of complex, context-dependent immunological data

**DOI:** 10.1186/1471-2105-7-341

**Published:** 2006-07-12

**Authors:** Randi Vita, Kerrie Vaughan, Laura Zarebski, Nima Salimi, Ward Fleri, Howard Grey, Muthu Sathiamurthy, John Mokili, Huynh-Hoa Bui, Philip E Bourne, Julia Ponomarenko, Romulo de Castro, Russell K Chan, John Sidney, Stephen S Wilson, Scott Stewart, Scott Way, Bjoern Peters, Alessandro Sette

**Affiliations:** 1 La Jolla Institute for Allergy and Immunology, 3030 Bunker Hill Street, Suite 326, San Diego, California, USA; 2 San Diego Supercomputer Center, P.O. Box 85608, San Diego, California, USA; 3 Department of Pharmacology, University of California, San Diego, 9500 Gilman Drive La Jolla California, USA; 4 Science Applications International Corporation, 10260 Campus Point Drive, MS-A2F, San Diego, California, USA

## Abstract

**Background:**

The Immune Epitope Database and Analysis Resource (IEDB) is dedicated to capturing, housing and analyzing complex immune epitope related data .

**Description:**

To identify and extract relevant data from the scientific literature in an efficient and accurate manner, novel processes were developed for manual and semi-automated annotation.

**Conclusion:**

Formalized curation strategies enable the processing of a large volume of context-dependent data, which are now available to the scientific community in an accessible and transparent format. The experiences described herein are applicable to other databases housing complex biological data and requiring a high level of curation expertise.

## Background

Many aspects of biological sciences have profited from the recent advances in the field of bioinformatics. New computational methodologies and tools allow researchers to capture, store, analyze and model large volumes of data, thereby dramatically affecting the pace, depth and scope of scientific investigation. A prerequisite for computational analysis is the availability of experimental data in an annotated, machine-accessible format. In research areas such as genomics and proteomics, such databases are a necessity, simply due to the vast amount of data generated. In the field of immunology, the majority of data are only reported in the literature, due to the typically smaller amounts of data distributed over many publications and to the dynamic and complex nature of immunological interactions. Thus, accurate representation of these data in a formalized fashion presents unique challenges.

Databases such as the International ImMunoGeneTics information system (IMGT) [1], AntiJen [2], Functional Immunology (FIMM) [3], HLA Ligand [4], SYFPEITHI [5] and the HIV database [6] house immunologically relevant information. They contain immunoglobulin-specific (Ig) resources, T and B cell epitope sequence data, and/or MHC binding data from peer-reviewed publications. Similarly, the Protein Data Bank (PDB) [7] functions as a service and repository for structural data and associated metadata of immunological relevance. While these databases are comprehensive in their respective scopes, they do not capture detailed, functional and contextual information related to immune epitopes in a structured manner. One exception is the HIV database, which contains detailed contextual descriptions of epitope recognition; however, this information is captured as free text and therefore, is not easily accessible for bioinformatics analysis.

To meet these challenges, the Immune Epitope Database and Analysis Resource (IEDB) was established as a centralized repository of immune epitope-associated information and related analysis tools [8,9]. It contains data on epitope recognition by the antigen specific receptors of the immune system, such as the structures bound by the T cell receptor or antibody as well as epitope related data on the interaction of an MHC with its peptide ligands. Knowledge regarding epitope structure and the immune response is important for the development of techniques that detect, monitor, and prevent or treat diseases.

The scope of the IEDB incorporates critical features related to the development of immune responses, such as data relating to the host, the organisms targeted by the immune response, and the biological context in which the interactions take place. Epitopes recognized by humans as well as all other host species are included, with current priority given to those derived from the National Institute of Allergy and Infectious Diseases (NIAID) Category A, B, and C priority pathogens, emerging and re-emerging diseases.

This is accomplished through modeling immune epitope data in an original and highly customized database utilizing a specialized ontology, encompassing over 300 distinct data fields, and organized into various classes and subclasses. These classes include epitope associated structural and source data, as well as contextual data such as MHC binding, MHC ligand elution, and B and T cell response data. Each class then contains a number of distinct fields, describing for example, the nature of the immunogen or antigen involved, the immunization route and schedule, and the assay type. A more detailed and technical description of the IEDB's structural ontology has been recently reported [10].

We here describe the design and implementation of a curation process to manage large volumes of context-dependent epitope-specific immunological data. The complexity of these data precluded full automation of the process and instead demanded sophisticated manual curation and the participation of subject matter experts. The necessity to capture a large volume of data in an accurate and consistent manner, as well as to provide transparency to the scientific community, required the development of a formalized approach to curation. Our results have general implications for the curation of complex, context dependent biological data, a prerequisite for managing the ever expanding knowledge generated in biological research.

## Construction and content

### Reference selection

The first step in our curation process is the selection of relevant articles. This is accomplished through a search performed on the entirety of the over 16 million citations in PubMed using a complex and comprehensive query specifically constructed for this purpose. The query consists of 61 keywords and logical operators to select epitope-related references [see Additional file 1]. Keywords such as "epitope", "major histocompatibility complex", "mimotope", "antibody", and "HLA" are applied to select for immune epitope related references in combination with a keyword filter to limit the selection of references by publication date, language and publication type, excluding review articles, editorials, meta-analyses, and comments. The query was modified with the input of subject matter experts who reviewed the results to ensure inclusion of all relevant references. As of January 31^st^, 2006, the query resulted in the selection of 96,565 references.

**Figure 1:**
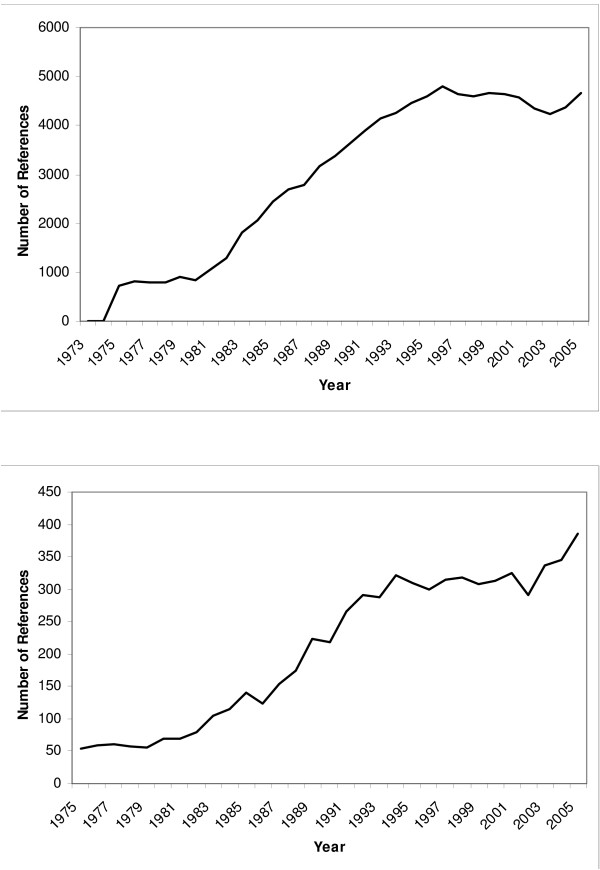
**Epitope related references present in PubMed. **a. References related to epitopes derived from all sources. b. References related to epitopes derived from NIAID Category A–C pathogens.

Figure 1a presents the number of potentially relevant references identified by the year of publication. Few immune epitope related references exist in PubMed before 1975. A rapid increase in relevant articles was observed during the late 1980s and then became steady at the current rate of approximately 4,600 new references per year. Of the identified references, 6,954 references also contained keywords associated with category A–C pathogens, a high priority as established by the NIAID. Publications relevant to epitopes from these pathogens began to appear in the 1970s with significant growth seen throughout the 1990s. A further increase in the number of A–C related epitope records is apparent from 2002 onwards, likely due to heightened attention given to biodefense research (figure 1b).

Because the search criteria were designed to be highly inclusive, some of the retrieved references are not actually relevant to the scope of the database and further manual review is required. After selection by the automated query, abstracts are formally reviewed for their relevance to the IEDB scope. Of the query selected references relating to the category A–C pathogens, 1,947 (28%) were deemed relevant to the scope of the project. Applying this same ratio to the broad set of 96,565 epitope-based articles, we estimate that a total of 27,000 articles currently exist in PubMed with potentially curatable epitope-related data.

As described above, the selection process for curatable references utilizes a combination of computer-based query and active evaluation by subject matter experts. We now have expert classifications for more than 11,000 abstracts into 'curatable' and 'uncuratable', generating a substantial dataset. Utilizing this dataset as a model, the process of manual abstract scanning and categorization can likely be automated using text classification tools tools. In a promising preliminary study of Latent Semantic Indexing, Naive Bayes Classifiers and Support Vector Machines classification techniques demonstrated that an automated approach could be applied to identify relevant abstracts.

### The curation and review teams

Capturing the complex context-dependent features of immune epitopes required the design and implementation of a novel approach to the data curation process. Few descriptions of manual data curation procedures have been reported in the literature [11,12]. In some cases, a flexible workforce of part time undergraduate staff has been utilized, but this strategy is inadequate when the data require considerable subject matter expertise. Large-scale involvement of experts can overcome this problem, as exemplified by the FANTOM project [13] and the initial phases of annotation of the human genome [14]. However, the ability to induce interest and commitment from large numbers of field experts is dependent upon the possibility of generating high impact publications in a relatively short time frame and is not sustainable in the long term.

Previous immunological databases have relied on close interactions between the immunological experts leading the database development and a limited number of dedicated curators, without the need for formalized processes to be developed. This approach has the advantage of producing high quality curation, but is not suited to large-scale curation of substantial amounts of data.

The complexity and volume of data within the scope of the IEDB project requires that manual curation be performed by a team of dedicated curators (the Curation Team; CT) with expertise in the areas of biochemistry, microbiology and immunology. Immunological expertise and independent assessment of the CT activities are provided by the Epitope Council (EC), an independent group of senior immunologists and structural biologists. In weekly meetings involving all CT and EC members, novel issues arising in the curation and review of specific references are discussed, together with curation rules, and work process issues. These meetings serve to educate new members through direct illustration of curation rules as applied to the references discussed. In this manner, a novel structure that efficiently integrates immunological experts and data curators allows the optimization of communication, curation consistency and continued training of the personnel involved in the project.

### Curation and review process

**Figure 2:**
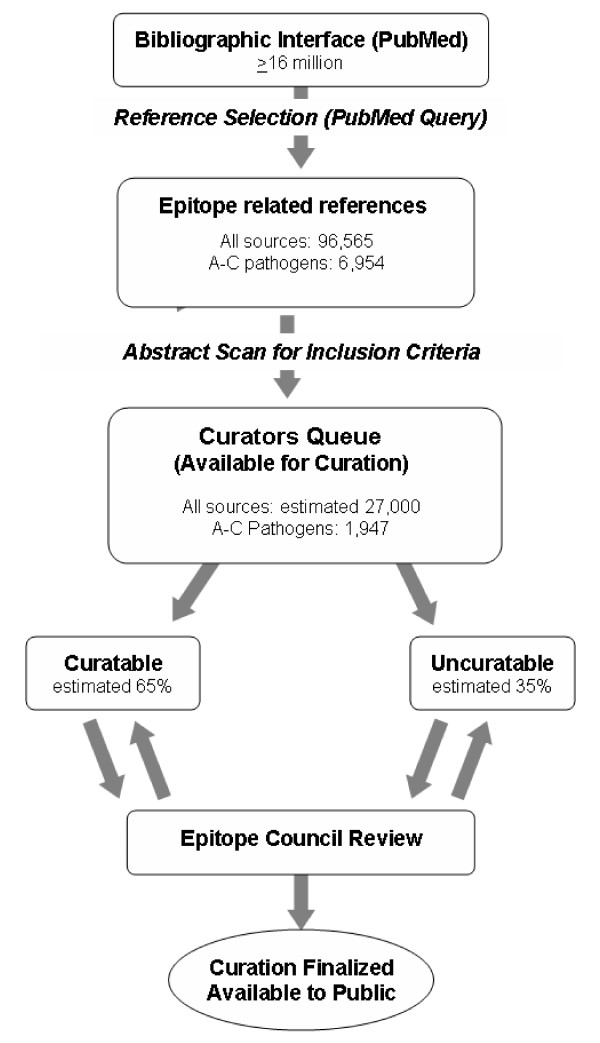
The IEDB curation process and statistics.

Electronic versions of the potentially relevant manuscripts are selected from a queue housed on an internal website and further analyzed by the CT. Because the initial evaluation of each manuscript is based solely on information contained within the abstract, the first step in the analysis is to assess whether the reference meets objective inclusion criteria. For example, our guidelines exclude references that do not include experimental immunological data, only relate to molecular structures larger than 50 amino acids in length or 5,000 Dalton in mass, use epitopes merely as tags for detection or purification experiments, or only report theoretical data without experimental validation. To date, approximately 35% of all references selected by query and initially approved through abstract review have been subsequently categorized as uncuratable (figure 2).

Once the manual curation of a reference is complete, the manuscript, the curation report, and tracking information are sent to the EC for review. This process is designed to be highly interactive and often requires the alteration of existing curation or "recuration" of the data prior to approval. Once approved by the EC, the curation item is promoted to the IEDB production site where it is then available for the scientific community. All internal site transactions and comments regarding the curation are retained for tracking, future referral, and educational purposes.

### Curation guidelines

**Figure 3:**
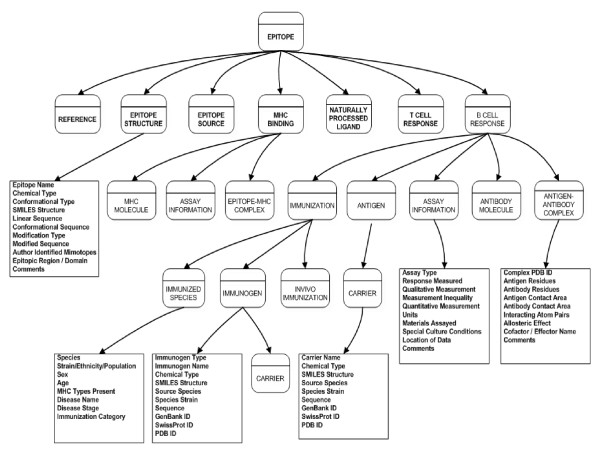
Relationships between an epitope and contexts.

The main challenge in the curation process is the accurate translation of the information contained in the literature into the structured format of the database. Epitope-specific interactions and assays are categorized by their immunological *context*, which refers to MHC binding and natural MHC presentation, as well as T cell and antibody interactions. For each interaction or assay, the context is specified by fields describing concepts such as the experimental MHC molecule, antibody type, immunogen, immunization procedure, carriers or adjuvants utilized and the type of assay performed with the qualitative and/or quantitative results. To illustrate the complexity of the relationships found in epitope-related data, figure 3 depicts example fields present in contexts such as epitope structure, immunization, and B cell response. The formal ontology of the IEDB provides the platform upon which the curation is based. A more detailed report of this ontology was recently published [10].

The nature of the data encountered in the literature led us to develop a Curation Manual and Data Dictionary to ensure the validity and standardization of the curation process. In these documents, rules, definitions, and guidelines were established to provide instructions regarding the strategies and procedures for capturing, annotating and introducing data from the literature into the IEDB. Although curation manuals of similar content are available for other immunological and biological databases [12,15-18], the specific relationships and complex nature of immune epitope data required novel guidelines of an explicit nature. For flexibility and accuracy, these guidelines are continually updated as new scenarios are encountered in the literature. Consensus is sought from the CT, EC and external subject matter experts prior to implementation. In this way, complex experimental data are captured in a consistent and accurate manner. The Curation Manual and Data Dictionary may be viewed on the IEDB website. In addition to serving as an internal tool for training and curation, the public availability of the Curation Manual may facilitate external submissions to the database, as well as ensure the transparency of the curation process and the criteria utilized by the IEDB staff to the scientific community at large.

The definitions established within the Data Dictionary are incorporated throughout the database. The system will display the definition and relevant information from the Curation Manual for a given field in the form of hints and on-line help.

### Computer assisted curation

As described above, the Curation Manual and the ontology of the database provide guidelines for the identification and manual curation of relevant epitope data. In order to enforce these guidelines and maximize curation accuracy as well as efficiency, the database contains a number of curation tools. One such application is the use of 'finders', which are widely utilized throughout the IEDB. Finders translate curator input into an established and controlled vocabulary as maintained in external databases focused on related and partially overlapping knowledge domains. For example, the source species and strain from which an epitope was derived are entered through the use of the *Species Finder *application, reflecting the established National Center for Biotechnology Information (NCBI) taxonomy. Through the use of synonyms, diverse terms present in the literature are mapped into a controlled vocabulary. Thus, the use of a shared vocabulary increases curation speed and data consistency, and connects the information contained in multiple databases. A similar effect is achieved for fields with a limited number of valid values through the use of drop down menus. The values present in these menus are subject to review by subject matter experts and are capable of evolution in parallel with the literature.

Curation is also assisted through inter-field validation, performed through automatic filling of linked fields. Essentially, certain fields dictate higher order concepts, allowing the system to fill select subfields. For example, the field values describing the type of assay antigen will be automatically filled by selecting *Epitope Source Species *as the assay antigen. In this case, the information previously entered manually describing the source species of the epitope will be entered into the fields describing the assay antigen. Additionally, copy capabilities simplify curation and minimize curator error. Entire epitope records, including experimental contexts, can be copied. Such copies may then be altered to correspond to a different epitope, allowing for efficient curation of data from references with multiple epitopes.

### Outcome curation

In order to simplify the curation process and accommodate large volumes of similar data within a given reference, the concept of outcome curation was developed. Outcome curation applies to the use of one immunological context to capture a number of similar experimental assays presenting the same outcome. For example, data describing the use of varying immunogen dose and resulting in the same qualitative outcome may be captured as one context entry. In order to minimize the potential for loss of detailed data, this strategy is governed by strict guidelines. This concept is only applied to multiple assays in which the variable is of limited consequence, such as dose or incubation duration, and not to multiple assays with critical differences such as the MHC allele or host species. Thus, the significance of all of the data is captured in an efficient manner. Outcome curation provides an effective strategy for enhancing the IEDB from both the curator's perspective, as well as the end user's perspective. It enables curators to capture a large amount of relevant data in a single representative entry in order to accelerate the curation process while preserving the richness of context-dependent information in an objective fashion. Furthermore, outcome curation offers benefits to the end user in the form of concise, non-redundant data.

### The curation tracking system

The curation of a large number of references by a team of curators and reviewers required the development of a formal tracking system. This specialized system allows formal tracking of the progress of curation from selection of the manuscripts until approved promotion into the IEDB, or finalization as uncuratable. The assignment of the reference to a member of the CT, the initial completion of curation, the assignment of the curation to an EC member for review, and the final approval of each curation are tracked. In this way, the progress of curation is monitored in real-time. Due to the large number of references relevant to the scope of this project, the tracking system has proven to be an invaluable tool in the management and development of the database, facilitating performance evaluation and identification of impediments to allow efficient reallocation of resources. Furthermore, the tracking system allows close monitoring of progress towards set goals and quick and effective direction of curation efforts towards high priority targets, as recently demonstrated by the complete curation of all references relating to Influenza A epitopes (Bui et al, manuscript in preparation) in a relatively short period of time.

**Figure 4:**
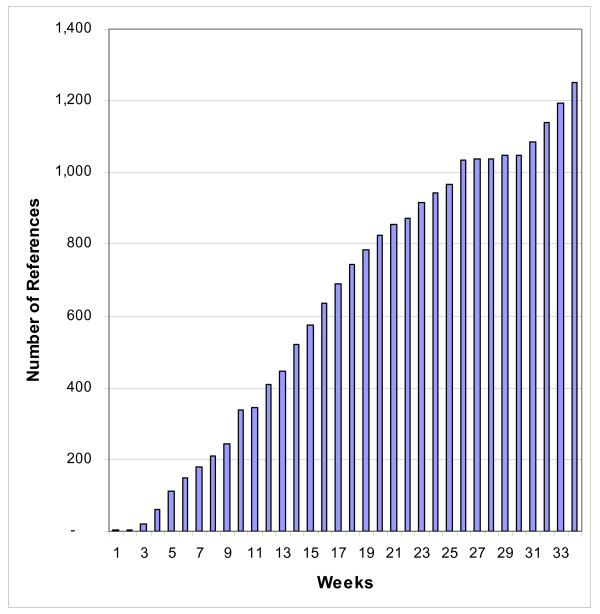
Total references processed as of February 5^th ^2006.

The processes and strategies described in the present report were developed over the course of the last year. Figure 4 depicts the output of finalized, manually curated references, including the entry of all distinct molecular structures and relevant experimental contexts over a period of 34 weeks starting June 13^th^, 2005. In all, over 1200 references were manually curated, with the rate of papers/week increasing in the latter part of the year, reflective of process optimization and the experiences gained by the CT and EC groups. Curation output slowed during weeks 26–30 due to transition from an internal system into a web-based curation site, performed over the holiday season. In accordance with the prioritization of the A–C pathogens, the curation of about 60% of the references pertaining to these pathogens has been completed.

The number of molecular structures captured is 12,201 with the number of distinct epitopes, defined as molecular structures associated with positive data for at least one context, at 8,144 as of March 2006. These results give an average of 12 structures per curatable reference examined, thus indicating that references frequently contain data related to more than one epitope or molecular structure and the same epitope may be reported by more than one curated reference. The corresponding total number of individual epitope contexts or assays curated is 27,431 or 3.4/epitope. These figures underscore how the IEDB curation strategy allows for the manual extraction of contextual information from immunological literature in an efficient manner.

## Utility and discussion

A novel process designed to capture complex and context-rich epitope data derived from relevant scientific literature has been presented. This process is multi-step, involving an automated query, a manual abstract scan to select potentially relevant references, followed by methodical analysis of the selected references, and finally the manual curation of papers deemed relevant to the scope of the database. This process controls quality, consistency and uniformity through an interactive review of the curated data by immunological experts and the development of a formalized set of curation rules. The selection of relevant references and tracking of the curation progress for a large number of references has been documented. Finally, tools to aid the curation process, such as finders, inter-field validation features and copy capabilities have been described.

Based upon the development of an optimized curation procedure, we have been able to process and finalize over a thousand references within a six-month period, with a trend towards further increase in curation speed. At our present pace, capturing all references containing immune-epitope related data will be completed within the next 5 to 10 years, depending upon the use of automated strategies and the size of the curation team.

The quality of the data captured by the IEDB has been further strengthened through continued recuration efforts. As new experimental scenarios are encountered in the literature, the rules applied to curation are expanded. On an on going basis, feedback from external experts in the fields of immunology and infectious diseases is sought in order to improve both the database structure and the curation practices. The curation tools and all fields and values utilized by the database are undergoing continual reevaluation and expansion. Improvements in all areas are retroactively applied to previously curated data in order to increase the sophistication of all data present within the IEDB. That it is impossible to anticipate all variations of data that can occur before the implementation of curation strategies represents an important lesson and illustrates the need for flexible design in curation systems handling such complex data.

The high quality achieved through manual curation compromises the speed at which data can be added to the database. The growing field of text mining may offer great utility to our reference selection process and to curation itself. The concept of automated field population is being embraced by the bioinformatics community with the development of numerous text mining programs such as Textpresso [19] and PreBIND [20], automated text recognition [21,22] and semi-automated annotation approaches [23,24]. These programs have the potential to increase the speed and accuracy of curation through automatic selection of relevant data and completion of database fields. However, these approaches are best suited to largely context independent data, and therefore not readily applicable to complex immunological relationships requiring interpretation of intricate experimental contexts. In the future, the large set of curated data generated by the IEDB could be utilized as a training tool for an appropriate text mining tool to attempt automation of the curation process itself.

## Conclusion

Increasingly, the process of scientific discovery depends upon the efficient retrieval of primary and secondary information from large databases storing highly sophisticated and annotated data. The processes involved in the creation of such complex databases determine their success as research tools. While some of the procedures described herein are undoubtedly utilized by other scientific databases, this information is difficult to access as details of the procedures used for in-depth literature-based curation are not generally available. Although our observations may be somewhat specific to our database, the lessons learned and the processes established during the implementation of the IEDB can be applied to the curation of experimental data relevant to other research areas presenting similar challenges. Thus, we hope that our experiences might be of some use to the developers and users of projects capturing complex and context-dependent non-immunological data as well.

## Availability and requirements

Consistent with our goal to optimize the manual and automated curation of complex data, encourage transparency of the associated processes, and facilitate integration between distinct databases, the IEDB project, together with the relevant code and documentation, are freely available to the scientific community .

The National Institute of Allergy and Infectious Diseases (NIAID) Category A, B, and C priority pathogens, emerging and re-emerging diseases list is available at: .

## Authors' contributions

RV, KV, LZ, NS, BP, AS, WF, HG, MS, JM, HHB, PEB, JP, RdC, RKC, JS, SSW, SS, and SW have been involved in drafting the manuscript or revising it critically for important intellectual content.

All authors read and approved the final manuscript.

## Supplementary Material

Additional file 1 Supplementary Figure 1. Query used to identify epitope related references. Click here for file
