# Supplementary material for: Curation of complex, context-dependent immunological data
Source: BMC Bioinformatics. 2006 Jul 12;7:341. doi: 10.1186/1471-2105-7-341 (PMC1534061; doi:10.1186/1471-2105-7-341)
Supplement: Additional file 1 — Supplementary Figure 1. Query used to identify epitope related references. [file 1471-2105-7-341-S1.doc]

**Query Parts**

1. Epitope Keywords

2. Filters

3. NOT keywords

(epitope[TW] OR epitopes[TW] OR mimotope[TW] OR ((MHC[tw] OR "major histocompatibility complex"[tw] OR HLA[tw]) AND (peptide[tw] OR peptides[tw])) OR "TCR recognition"[tw] OR ("Class"[tw] AND "I motif"[tw]) OR supermotif[tw] OR immunogenic linear OR ("peptide-based"[tw] AND CTL[tw]) OR phage displa*[tw] OR "antibody binding"[tw] OR "protective immune response"[tw] OR antibody recog*[tw] OR "cytotoxicity assay"[tw] OR "new monoclonal"[tw] OR "novel antibody"[tw] OR ( (monoclonal antibod*[tw]) AND "binding site"[tw]) OR ( (KA[tw] OR KD[tw]) AND (monoclonal[tw] OR mAb[tw])) OR "neutralizing antibody"[tw] OR "peptide vaccine"[tw] OR (peptide conjugate vaccine*[tw]) OR ((CD8[tw] OR CD4[tw]) AND “T cells”[tw] AND (peptide[tw] OR peptides[tw])) OR (“antigenic repertoire”[tw]) OR ((peptide[tw] OR peptides[tw]) AND “antibody reactivity”[tw]) OR ("Class II"[tw] AND (binding [tw] OR bound[tw] OR peptide[tw] OR peptides[tw])) OR "immunogenic peptide"[tw]) **AND** (hasabstract[text] AND English[Lang] AND ("1900"[PDat]:"2006/02/28"[PDat])) **NOT** (Review[PT] OR Editorial[PT] OR meta-Analysis[PT] OR Comment[PT])
